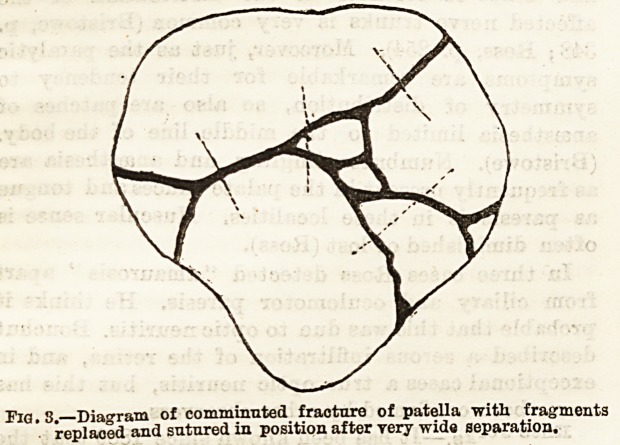# The Treatment of Fractures of the Patella

**Published:** 1896-02-22

**Authors:** John Poland

**Affiliations:** Visiting Surgeon to the Miller Hospital, Surgeon to the City Orthopædic Hospital, and Consulting Surgeon to the St. Pancras and Northern Dispensary, &c.


### MEDICAL PROGRESS AND HOSPITAL CLINICS.

[*The Editor will be glad to receive offers of co-operation and contributions from members of the profession. All letter should be addressed to* THE EDITOR, AT THE OFFICE, 428, STRAND, LONDON, W.C.]

### THE TREATMENT OF FRACTURES OF THE PATELLA.

By JOHN POLAND, F.R.C.S., Visiting Surgeon to the Miller Hospital, Surgeon to the City Orthopædic Hospital, and Consulting Surgeon to the St. Pancras and Northern Dispensary, &c.

(*Continued from page* 331.)

A more simple operation than these has been introduced by Mr. Mayo Robson, and is as follows: The limb is carefully washed and sponged with perchloride or carbolic lotion. After aspiration of the joint, two long steel (bonnet) pins with glass heads are then thoroughly purified and passed, one from without inwards through the quadriceps tendon

FIG. 2.—Mayo Robson's method of using pins in simple transverse fracture.

immediately above the upper fragment, and made to project on the opposite side of the knee at a point corresponding to the point of entry; the second in like manner through the upper end of the ligamentum patellæ just below the fragment. By gentle traction on the pins the fractured surfaces are brought into contact, and a figure of eight suture of aseptic silk or whipcord is passed over the projecting ends of the pins on either side (not over the patella). The projecting ends of the pins are then cut off and antiseptic gauze applied over the punctures with an immovable apparatus. This plan (Fig. 2) seems to be at once simple and effectual without opening the joint. There are many modifications recommended by Ceci, T. G. Morton, Volkmann, Marshall, T. G. Twynam, A. H. Butcher, and many others too numerous to be mentioned. The two last mentioned are excellent methods of circumferential suture, and used by many surgeons. The interposition between the fragments of shreddy portions of the extensor tendon and capsule is a frequent source of trouble, and in many these fibrous and aponeurotic tags are so tightly applied between the fractured surfaces that they offer an insurmountable obstacle to the exact apposition of the osseous surfaces. This fact, together with the frequency with which so-called sub-cutaneous or extra-articular suture leads to opening of the joints, has induced many to discard entirely these methods of treatment. Whatever the plan of treatment, a firm, accurate, and short union should be aimed at, even though it be accompanied by a more or less stiffened condition of the knee. The immovable splint should be kept on for six weeks. After this the patient may get up and walk about on crutches for the next three or six months with the knee joint constantly supported. Any sudden muscular movement that may stretch the soft uniting union must be guarded against. Gentle massage to the joint and extensor tendon of the thigh will be found exceedingly useful in shortening the period of complete recovery and in freeing the articular surfaces and muscles of adhesions and strengthening the weakened quadriceps.

Passing now to the last method of treatment, viz., by exposing the fragments, under strict aseptic and antiseptic precautions, and wiring or suturing them together. This plan was reintroduced in this country by Sir Joseph Lister in 1877. He soon had many followers, especially in France, Germany, and America. There are now some surgeons who advocate suturing in every recent case, while others decry the operation altogether on account of the dangers attending the opening of so large and important an articulation. There is a risk and danger to the patient, no matter how carefully the operation is carried out. Hamilton said, "I wish to add my own testimony, that it is offering a very grave and dangerous substitute for others perfectly safe, and, so far as is yet proven, equally efficient methods; it is hazarding the life of the patient without offering any equivalent. Indeed, I do not see why anything less could be reasonably expected from this kind of surgery than tedious recovery, anchylosis, amputation, or death, at least in a considerable proportion of cases, and this is precisely what has happened." Again, Von Bergmann said, "Whatever faults may be charged against the unsuccessful surgeons and their antiseptic measures, the fact of frequent failure cannot be disregarded, and all the more because the majority, the very great majority, of transvere fractures recover with relatively good restoration of function without suture and without operation, even when the reunion of the fragments is not close and bony." Recent aseptic and antiseptic measures have done much to modify these decided opinions; yet, recognising the risks of the operation, one cannot help agreeing largely with them, and feeling that, like many novel surgical operations, it has been employed in the past far too indiscriminately. Patients have been subjected to serious operations who might have been treated quite as successfully by milder measures—that are not inclined to expose them to grave dangers to limb and even life, which far outweigh the loss of six months, serious though it be to a working man. With regard to the subsequent utility of the limb, is it better for the patient to go about with a stiff knee, close apposition of fragments, and a firm limb, with the possibility of gaining more freedom of movement in time by use, &c., or to have a weakened condition of extensor muscle, with wide separation, and the chance of breaking other bones and becoming permanently crippled? Again, with what amount of separation after the different forms and degrees of severity of fracture can a patient carry out his particular vocation? These questions are not so easy to answer as they may at first sight appear. It is exceptional in hospital practice to see patients with fractured patella after the lapse of years. They are occasionally admitted again for some other affection. Sometimes the very wide separation of the fragments produces a crippled condition of the limb, and leads to fracture of the other patella, of the thigh, leg, or other bone. A good example of this came under my care not long ago. A man, aged fifty-nine, had ten years previously fractured his left patella, for which he received no treatment. As a result, there was a crippled condition of the limb, with three inches or more separation between the fragments. He was continually falling down in consequence; at one time he fractured his left leg severely, and then his right patella, for which he came under treatment. I have lately seen another case where the femur of the same side was fractured through the same condition of the limb. Even with a certain amount of separation the limb is an exceedingly useful one for ordinary purposes. By gradual stretching of the uniting material extending over years, it is surprising that the separation, though great, does not in some instances prevent the patient from following a laborious occupation. In the discussion before the Clinical Society in 1883 the operation of wiring was warmly advocated by Sir J. Lister and others. At that time I was surgical registrar to Guy's Hospital, and at Mr. Bryant's suggestion collected thirty-two cases of fractured patella which had been admitted into this hospital for some other reason during a period of sixteen years. The following are a few of them:—

| Age. | Period since Fracture. | Separation. | Remarks. |
|---|---|---|---|
| 64 ... | 34 years ... | 4 inches | ... Able to work well as cellarman. |
| 37 .. | 1 year ... | 2 inches | ... Weak, but able to work well. |
| 63 ... | 2 years ... | Wide | ... Able to work well. |
| 29 ... | 9 mths. ... | Wide | ... Able to work as a groom. |
| 53 ... | 14 years ... | Wide | ... Good worker. |
| 47 ... | 6 years ... | 2 in. both sides | ... Walks well. |
| 52 ... | 1 year ... | 3 inches | ... Good limb. |

Many others with short union, *i.e.*, ¼ to ¾ in. separation, were able to work, many as well as ever. They are examples of what should be the result of all ordinary simple cases of transverse fracture. Hence, it is obvious that a certain proportion of patients are able to follow their laborious occupations after they have stretched the uniting material of their fractured bones; while there are an immense majority that cannot be treated better than by the methods given above. But, on the other hand, a close union of the fragments does not always give the most useful limb, therefore it is questionable whether we should convert by a surgical procedure an injury which, in a large number of cases, may be treated by simple plans and without danger, into one that is not devoid of risk even to life. An attempt ought to be made to discriminate those that may incur the extra risk, be suitably operated upon, and with benefit.

The text-books state that simple transverse fracture of the patella is nearly always caused by muscular action. My own experience is somewhat different. After carefully inquiring of patients into the history of the accident in a considerable number of cases admitted into Guy's Hospital during the years I was surgical registrar at the hospital, I found that more than a third of all cases of transverse fracture were caused by direct violence, combined with the muscular action. Subsequent experience has corroborated this opinion, and a French surgeon has expressed the same view. When the knee is slightly flexed by muscular action, and the patella placed on the condyles of the femur, a slight amount of violence applied to the front of the bone is sufficient to cause it to fracture, and to be accompanied by very serious lesions. The powerful extensor muscle continuing to act upon the upper fragment and sides of the patella, the capsule and the rest of the aponeurotic coverings causes most extensive laceration of the capsules, ligaments, and aponeurotic layers over the front and sides of the joint with considerable extravasation of blood into and around the joint. The remains of the extensor tendon and tags of fibrous tissue, which are tightly drawn in between the fragments and cover the fractured surfaces, and the large amount of clotted blood entangled in these aponeurotic tags and in and around the articulation render it a matter of impossibility to bring the fragments into apposition. If in these exceptional cases there is the history of the accident I have indicated, and when in consequence there is strong presumptive evidence of the existence of the above lesions, an open operation should be performed under strict aseptic precautions, otherwise a more or less crippled or useless limb is sure to result. I have under these conditions performed Lister's operation on several occasions, with most satisfactory results in each case. In 1889 I had a very successful example of wiring the patella. Although it was an unusual case of comminuted fracture, caused by direct violence, that this was combined with muscular action was indicated by the very great separation of the fragments. The condition of the articular and periarticular structures was very similar to those just described. Beyond this there existed very wide separation between the numerous fragments. The following is a brief note of the case:—

The patient, a brewer's drayman, aged twenty-nine, fell a distance of thirty feet, his left knee coming in contact with the stone pavement. His fall, however, was broken before reaching the ground. Very considerable swelling about the joint obscured the precise condition of the fractured patella. At the time of the operation it seemed at first impossible to bring the fragments of the bone together, on account of the extensive comminution, the wide separation of the fragments, and the difficulty experienced in immediately recognising the proper position of the various pieces. This was ultimately accomplished by drilling the fragments and drawing them together by means of sutures of pure silver wire of small size. The patella then moved as

Fig. 3.—Diagram of comminuted fracture of patella with fragments replaced and sutured in position after very wide separation.

a whole. (See Fig. 3.) The joint was syringed out with a strong solution of carbolic acid. The torn extensor tendon, capsule, and vasti aponeuroses were finally united over and at the sides of the patella by means of fine silk sutures, for I look upon the union of these fibres as a most important factor in obtaining rapid and firm consolidation of the fragments. The wound was dressed on the second day, when the temperature rose to just over 100; after that, at intervals of about every fourth day, the temperature remaining normal. The patient left the hospital at the end of eight weeks with a plaster of Paris splint. Six weeks later a small sinus formed, through which one of the wires was easily removed; the rest remained in position. At the end of twelve months the patient could walk well without the aid of a stick or support of any kind, and flex his knee to some extent. The patella appeared to be one solid osseous mass, without a trace of separation between the fragments. Dr. E. M. Cox, of New York, uses ("Annals of Surgery," Dec., 1895, p. 725) catgut sutures, and washes the joint clean with physiological saline solution. Dr. G. R. Fowler, of Brooklyn, has recently ("Annals of Surgery," June, 1895) recommended a new operation, which consists essentially in exposing the fragments as an intermediate procedure, *i.e.*, after the immediate effects of the injury have subsided and before the occurrence of ligamentous union, for the purpose of clearing their surfaces of intervening soft parts, and the application of fixation-hooks, somewhat resembling Malgaigne's, except that a single instead of a double pair is employed. The hooks are permitted to remain for about three weeks; they may be reapplied if the union be not complete or firm. This method has no advantage, except its simplicity, over the use of suture, but it demonstrates well that the fragments can be held together without much strength, and that only one wire is necessary in many instances of Lister's operation, and that it may even be replaced by silk.

Compound fracture of the patella is now successfully treated by wiring or suture with strict antiseptic measures, saving many limbs and lives which were formerly lost by pyæmia, exhaustion, or amputation. In the case of a boy with compound fracture of the patella complicated by other fractures, Dr. E. M. Cox recently succeeded ("Med. Record," April 27th, 1895) in obtaining a perfect result. The restoration of function was complete. Catgut was used for the sutures. I quote this as an example of many others treated in the same way. A detailed analysis of a series of eighty-four cases of this injury with the treatment adopted at that time is to be found in the "Roy. Med. Chi. Trans." for 1870 by the late Mr. Alfred Poland. It affords an interesting comparison with the results of modern surgery a quarter of a century later.

To conclude by way of summary:—

(1) In all cases of fractured patella the fragments must be closely approximated—the shortest possible fibrous or osseous union should be our object.

(2) In the most simple cases of transverse fracture and stellate or vertical fracture from direct violence this may be effected by a simple immovable apparatus.

(3) In simple cases of transverse fracture with effusion of blood or fluid aspirate at once, and apply a suitable immovable splint to retain the fragments in close apposition.

(4) Or after aspiration employ Mr. Mayo Robson's pins, fixing the limb immovably.

(5) Operation by incision and wiring or suturing in exceptional cases after direct violence combined with muscular action, in which great destruction of the articular structures, with hæmorrhage into and round the joint, are believed to be present. In comminuted fractures from direct violence with separation of the fragments and in compound cases.

The functional disorders to the limb consequent upon old fractures of the patella have not been considered in this paper. Their pathology, mechanism, and necessary treatment by operation, &c., has been carefully studied by Dr. Choux in the "Rev. de Chirurg." for March 10th, 1894, to which I would refer those interested in the subject.

## Figures and Tables

**Fig. 2. f1:**
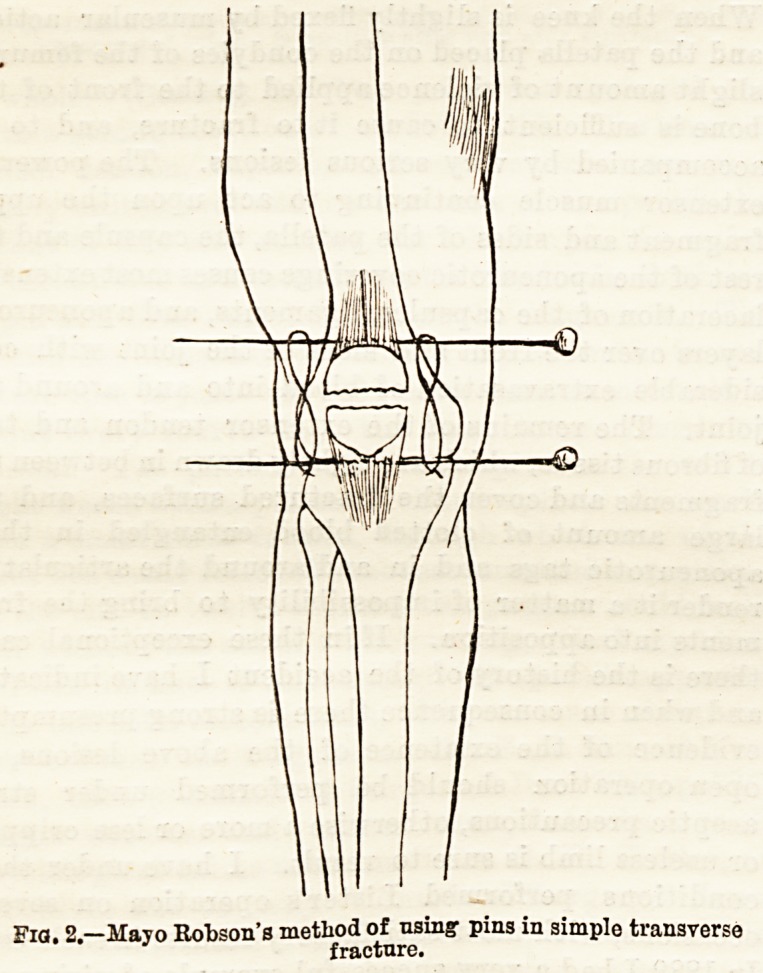


**Fig. 3. f2:**